# The Role of Sum-Frequency Generation Spectroscopy in Understanding On-Surface Reactions and Dynamics in Atmospheric Model-Systems

**DOI:** 10.1021/acs.jpclett.4c00392

**Published:** 2024-04-18

**Authors:** Clara-Magdalena Saak, Ellen H. G. Backus

**Affiliations:** University of Vienna, Faculty of Chemistry, Institute of Physical Chemistry, Währingerstrasse 42, 1090 Vienna, Austria

## Abstract

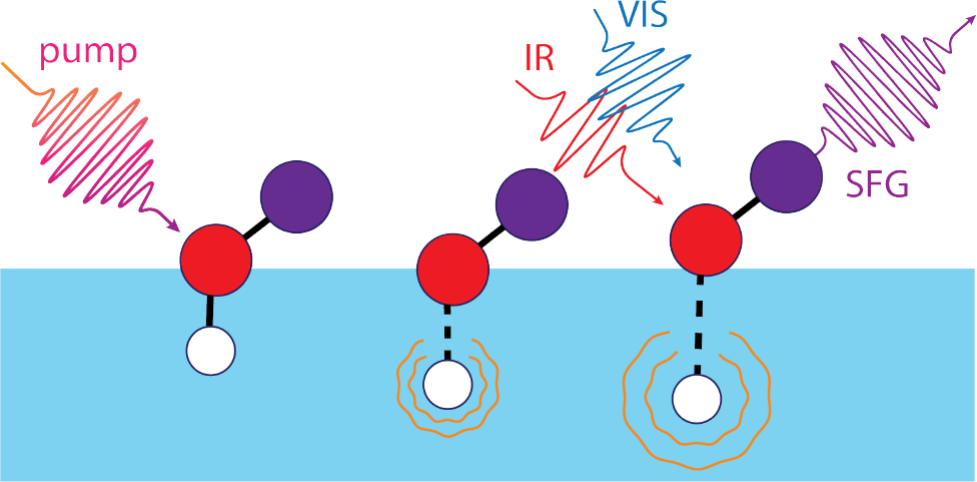

Surfaces, both water/air and solid/water, play an important role in mediating a multitude of processes central to atmospheric chemistry, particularly in the aerosol phase. However, the study of both static and dynamic properties of surfaces is highly challenging from an experimental standpoint, leading to a lack of molecular level information about the processes that take place at these systems and how they differ from bulk. One of the few techniques that has been able to capture ultrafast surface phenomena is time-resolved sum-frequency generation (SFG) spectroscopy. Since it is both surface-specific and chemically sensitive, the extension of this spectroscopic technique to the time domain makes it possible to study dynamic processes on the femtosecond time scale. In this Perspective, we will explore recent advances made in the field both in terms of studying energy dissipation as well as chemical reactions and the role the surface geometry plays in these processes.

The air/water interface is one of the most important environmental interfaces due to its prevalence and the diversity of reactions it facilitates in the atmosphere. While surfaces mediate processes in many different applications and fields, their properties are particularly important in atmospheric processes involving aerosols due to the large surface to volume ratio of these systems and their pronounced impact on net radiative forcing.

One of the most intuitive roles that surfaces play in the atmosphere is as a meeting point between the gas phase and the condensed phase, be it liquid or solid, and is thereby necessarily the site for any phase transition behavior, such as condensation or evaporation. Most prominently, the condensation of water and the growth of droplets during cloud formation come to mind or, inversely, the evaporation of moisture from large bodies of water. In particular, the growth and formation of cloud droplets from aerosol particles is a key process in understanding climate on a larger scale, since the connection between aerosols and clouds is one of the largest sources of uncertainty in current climate models. This balance of condensation and evaporation can be tipped to either side depending on the properties and composition of the surface, which mediates both.

Aerosol particles contain a large variety of chemical compounds, depending on their origin as well as the degree of aging, which changes the particle composition due to for example oxidation of organic compounds. However, even when the overall composition of a droplet is known, the dissolved compounds can show very different surface propensities. Understanding both the surface structure and the changes to the interface that happen in the presence of different compounds is central to understanding the roles they play in the atmosphere.

Unfortunately the direct spectroscopic study of the aerosol particle interface is challenging. Many surface sensitive spectroscopic techniques require vacuum conditions, which strongly affect the composition of the particle by evaporation. On the other hand, nonlinear optical and vibrational techniques have only recently been expanded to the scattering geometry and are affected by relatively low signal levels.

Due to these experimental challenges the flat surface is commonly used as a model system. The flat surface is considered a good description of the surface structure found in large particles (>1 μm); however, it is well-known that smaller submicron particles may exhibit behavior which is unique to their size. Such effects are clearly not captured by the flat surface.

However, the environment of the flat surface already gives rise to changes in the collective molecular and electronic structure, which are uniquely different from the well studied bulk, making it a necessary first step to understand the flat surface before the droplet may be fully characterized. With the advancement of scattering techniques in nonlinear spectroscopy and ambient pressure electron spectroscopy, hopefully new experimental opportunities to study both static and ultrafast processes directly on the particle interface may arise.

The air/liquid interface has been subject to extensive research using a multitude of techniques,^1,2^ such as X-ray photoelectron spectroscopy (XPS),^3^ sum-frequency generation spectroscopy (SFG),^4,5^ second harmonic generation (SHG),^6^ X-ray and neutron reflectometry^7^ and many modeling approaches from classical molecular dynamics (MD) to ab initio.^8^ Most of these studies have focused on understanding the composition of aqueous surfaces and the enrichment of different common solutes as a function of their respective bulk composition. In particular, surface enrichment of small molecules and ions has been investigated in detail.^1^ The underlying driving forces of surface enrichment have been found to go beyond simple hydrophobic effects, as even highly soluble ions can be enriched at the surface.^9^ These changes in ion distribution, in turn, affect the hygroscopicity of the particle significantly, thereby changing its ability to act as a cloud condensation nucleus.

Here SFG has been particularly impactful due to its inherent surface selectivity and chemical specificity, which allows for the structural investigation of the water surface itself, but also of the surface composition in mixed systems. In this Perspective, we will focus on time-resolved SFG experiments, which can help elucidate the structure and dynamics that take place on atmospherically relevant surfaces.

Sum-frequency generation is a second order nonlinear process which, as the name suggests, involves the generation of light at the sum frequency of two incident beams which are overlapped in time and space at the sample surface. When the IR beam is in resonance with a molecular transition, the process becomes resonantly enhanced.

The intensity of the SFG signal is mainly determined by the second-order nonlinear susceptibility χ^(2)^, which can be understood to be the ensemble average of the molecular hyperpolarizability. As all even-ordered nonlinear processes, SFG is only allowed in noncentrosymmetric media under the dipole approximation. In the case of liquid water, a symmetry break is introduced by the formation of an interface. Therefore, only ensembles of molecules in the immediate surface region, which show a net orientation relative to the surface plane, can give rise to any SFG signal. In the case of vibrational SFG the frequency of the two beams is chosen such that one, usually a broad-spectrum IR pulse, is in resonance with a molecular vibration (see Figure 1). The second beam, usually in the visible range between 400 to 800 nm, is then used to upconvert the polarization induced by the IR field into a virtual state from which a photon is emitted, similarly to the emission of a Raman photon. The general SFG process is sketched in the top panel of Figure 1. A more detailed description of the process can be found in the literature, for example in ref (10).

**Figure 1:**
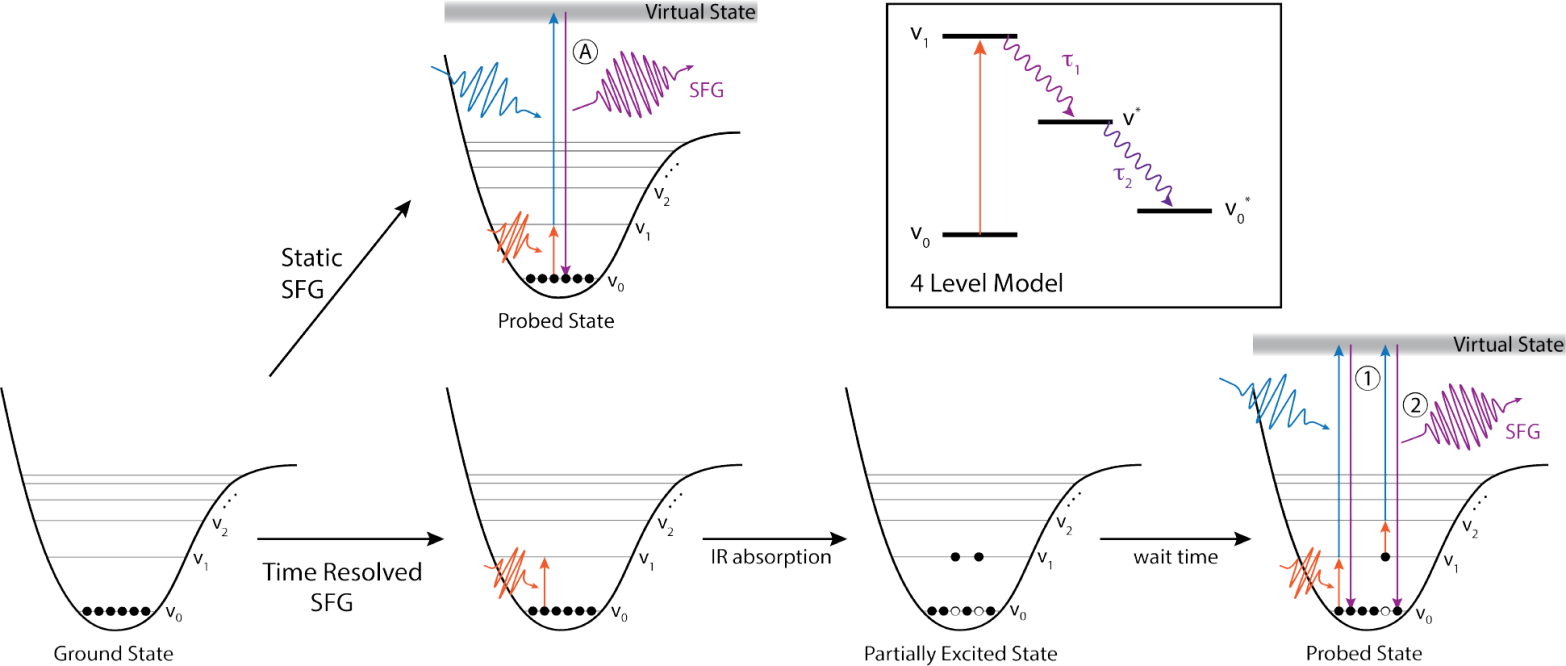
Sketch of the most relevant steps in obtaining static and time-resolved SFG. Top: unpumped/static SFG (A) marks the emission of SFG light from the vibrational ground state; in the absence of a pump pulse, the vibrational ground state is fully populated. Bottom: IR-pump SFG-probe Experiment - (1) marks the emission of light from the vibrational ground state, due to a reduced population of the ground state after interaction with the pump beam this feature is referred to as ground state bleach (GSB), (2) marks the emission of light from a vibrationally excited state, also referred to as excited state absorption (ESA). The insert in the top right depicts the 4-level model which is commonly used to describe the vibrational dynamics of the hydrogen bonded OH stretch region both in bulk and at the interface. ν_0_ and ν_1_ refer to the vibrational ground and first excited state. After excitation, the system first relaxes into a different excited state (e.g., a bend overtone) ν* before reaching the heated ground state ν_0_^*^, see main text for detailed description.

Another important aspect of SFG is that varying the polarization of the incoming and outgoing beams allows one to probe different components of χ^(2)^, which may in some cases allow for a determination of the average orientation of groups or to enhance vibrations of a certain symmetry.^10^ The combination of polarizations most commonly used is SSP, referring to S polarized SFG light, S polarized visible light, and P polarized IR light. Another combination that is often used is PPP. The choice of polarization, as well as the angles of the respective beams, will also impact the relevant Fresnel factors at the experimental surface. In general, the Fresnel factors describe the reflection and transmission of beams at a given interface and geometry. Since most SFG experiments are performed in reflection geometry, it can therefore be necessary to consider the relevant Fresnel factors describing the reflection of the beams. Especially when working with buried interfaces, it can be crucial to carefully choose the experimental parameters to efficiently probe the sample interface.

One disadvantage of SFG measurements as described so far (so-called homodyne SFG) is that the absolute square of the emitted SFG field, or in other words its intensity *I*_*SFG*_ = |*E*_*SFG*_|^2^ ∝ |χ^(2)^|^2^, is detected. This means that any information on the relative phase of the emitted light is lost. This can be disadvantageous since the phase is linked to the sign of the alignment of a group of oscillators, i.e. whether they are pointing up or down relative to the surface plane. This information can be recovered by so-called heterodyne SFG, where the SFG field emitted at the sample is interfered with the temporally shifted signal of a local oscillator. The resulting interference allows for the recovery of the imaginary part of χ^(2)^ and hence the up or down orientation of the molecules.^11−13^

Both homo- and heterodyne SFG have been expanded to include measurements in the time-domain. This involves the addition of a pump pulse preceding the SFG probe pair (and in the case of heterodyne SFG the local oscillator). Such an experiment using an IR pump is sketched in Figure 1. However, experiments may also involve a pump pulse in the visible or ultraviolet range, resulting in an electronic excitation. While there are subtle differences to the results obtained with time-resolved homo- and heterodyne SFG, the main observables are found to be very similar^14^ and thus, results on relaxation dynamics can be compared across the two techniques.

Another variation on the pump/probe scheme is the 2D experiment, which introduces a broadband pump pulse pair and allows for even more advanced studies. Such 2D experiments have been performed for vibrational pumping and SFG probing^15^ and even using a visible pump pair.^16,17^ In principle, these techniques allow the same type of information to be extracted as time-resolved experiments using narrow-band pump beams but at a wider range of frequencies in a single experiment.

Due to the often complex nature of both the static SFG spectrum, and especially the transient changes that appear in time-resolved experiments, SFG experiments often need to be supported by modeling studies to fully explain the underlying structural changes occurring. Such studies span the complexity of the computational field at varying levels of theory. For a detailed overview of the current state-of-the-art the reader is referred to the respective literature,^18−21^ with some examples given here studying SFG emission from water^22−28^ and ice^29^ surfaces. In this Perspective, we will focus on experimental studies and only discuss accompanying theoretical modeling where applicable for those specific studies.

Due to its surface selectivity, SFG has been widely applied in the context of environmental sciences, for example, it has been used to study the mechanism and likelihood of uptake of gaseous components at the water surface, such as methanol and acetone.^30,31^ These volatile organic compounds (VOC) heavily contribute to aerosol aging and growth. It has been shown that the absorption of VOCs is strongly dependent on the nature of the condensed phase, and even simple changes such as the freezing of water into ice can impact the way small organics interact with the surface of, for example, an aerosol particle. Cyran et al.^30^ have shown that while acetone readily adsorbed to both the ice and water interface, the molecular interaction between the liquid water molecules and the adsorbent is markedly different. The interaction of the organic with the ice surface is rather specific, mainly via an interaction with the free OH groups. It is, however, much less defined in the case of liquid water.

For a more detailed overview of the advances static SFG experiments have contributed to in the field of environmental sciences and in particular the understanding of air/liquid interfaces, the reader is referred to the work by Jubb et al.^5^

However, particles are often found to be more complex than simple single-phase systems, i.e., a fully solid or liquid particle which remains as such, but are composed of both solid and liquid parts, either in the form of core/shell or aggregate particles. These structures can form over long periods of time for example when water condenses onto a mineral dust or when salts effloresce at low relative humidities,^32^ making the overall morphology of the aerosol particle malleable as both the phase and composition of the particle are subject to frequent changes. This being said, it becomes clear that the air/particle interface is not the only interface we have to contend with but that the solid/liquid or even liquid/liquid interface may play just as large a role in determining the overall chemistry of the droplet in the atmosphere.

In particular, the mineral/liquid interface has recently been studied in more detail. These systems can hold particularly relevance to atmospheric systems,^33^ and small mineral particles are often taken up into the atmosphere^34^ as for example dust clouds and act as a nuclei for condensation and droplet growth. Prominently, we have seen clouds of Saharan desert sand tint the skies over European cities^35^ and dust and black carbon spread over large regions following wildfires,^36^ which highlights how far these particles can travel in the atmosphere.

The initial condensation of water onto aerosol particles is likely more akin to wetting phenomena and seeded condensation. However, once a significant layer of liquid is formed around an insoluble mineral core, the particle may now be considered as a core/shell particle. These combined phase particles feature two distinct interfaces that need to be considered, namely, the outer liquid/air interface and the internal solid/liquid. The solid/liquid interface is quite interesting because, depending on the properties of the liquid phase, it can develop quite a pronounced surface potential, which in turn is known to affect the orientation and hydrogen bonding of the water in close proximity.^37,38^ This suggests that at, for instance, varying pH or salinity of the aqueous phase the properties of the solid/liquid interface might vary quite strongly, which in turn may affect any dynamic processes occurring at the second interface in biphasic particles of this nature.

Beyond the considerations of physical transitions and mass transport, the aqueous surface plays a key role as the site for many chemical reactions. Most simply, the presence of a surface may affect the probability of the reaction between solvated molecules and the surrounding gas phase to occur, exposing surface enriched compounds to highly reactive components within the gas phase, such as ozone, while obscuring others within the bulk of the liquid.

It has even been suggested that the efficiency of some reactions may be enhanced at the aqueous surface,^39,40^ making it particularly important to understand the role of the surface in the reaction mechanism, and whether for example the energetic barrier of activation may be lowered at the surface. Therefore, the aqueous surface has to be regarded as a unique chemical environment with its own reactive landscape, distinct from that of the bulk, possibly leading to different reaction pathways being favored and time-constants to be altered.

The abundance of intense sunlight in the atmosphere makes photochemical reactions one of the most important chemical conversion pathways and also contributes to the continuously changing properties of aerosols and clouds. In order to better understand how these reactions affect the surface composition and the role of aerosols in different processes, it is first necessary to address the outstanding questions about the way surfaces can impact dynamic processes, ranging from the dissipation of vibrational energy and heat to light induced radical reactions. The latter being particularly important in the context of particle aging in the atmosphere.

Here we draw a distinction between two different types of processes that may occur when the surface is exposed to light, namely, photophysical and photochemical processes. Photophysical processes may include the relaxation/deexcitation after light absorption through, for example, vibrational relaxation, internal conversion, fluorescence, and phosphorescence but leave the chemical structure and composition unaltered. On the other hand, photochemical processes mainly involve the breaking of a chemical bond, which predominantly leads to the formation of radicals or the transfer of electrons from one molecule to another. The initial photodissociation can subsequently trigger a chain of radical reactions, depending on the nature of the parent molecule and the reactivity of the radical fragments.

Most work presented in this Perspective focuses on the OH stretch region at around 3200–3700 cm^–1^, depending on the specific chemical environment the oscillator experiences. This region is likely favored over other vibrational bands, such as the bend mode, due to its more intense SFG signal, which eases some of the experimental difficulty of performing these types of experiments. However, it has also been shown that the bend and stretch mode of liquid water provide slightly different information about the aqueous phase:^41^ the bend mode appears to be more localized on the individual water molecules, whereas the stretch mode attains more of a delocalized character in strongly hydrogen-bonded environments. The former can therefore be considered to report more on local properties of the water molecules themselves, and the latter on the properties of the hydrogen bond network the molecules are embedded in. Since the hydrogen bond network is key to understanding the behavior of water and is certainly a key player in any processes of energy dissipation, the choice of the stretch region as an experimental window may provide a more representative view of the role water plays in any processes studied.

The transfer of heat and excitation are relevant processes in reaction dynamics not only because the time scale of de-excitation is an important aspect of the reaction itself, but the lifetime of vibrational states can also give important information about the structure, i.e., how strongly molecules are coupled to each other. A strong coupling across, for example, the hydrogen bond network may even suggest that certain groups of molecules have to be considered as a delocalized system in the mechanism of different reactions.

Water acts as the solvent of most condensed phase atmospheric chemistry. Any excess energy that is either absorbed from solar radiation or produced during a reaction will have to be dissipated via the hydrogen bond network of water. Since clouds and aerosols can have both liquid and ice fractions, it is relevant to understand these processes in both phases.

Time resolved experiments make use of the deposition of vibrational energy into a specific subset of functional groups via an additional IR pump pulse preceding the SFG probe pair (Figure 1). The dissipation of this vibrational excitation can then be used to infer information about dynamic processes, such as structural rearrangement or intermolecular coupling.

The general principle of this kind of experiment at the water interface has not changed since the first work published by McGuire and Shen in 2006^42^ (the results of which will be discussed later in the context of solid/liquid interfaces). These experiments were then initiated to answer the question of whether the structural differences between bulk and surface (mainly the truncation of the hydrogen bond network) would lead to different dynamic behavior or if the remaining hydrogen bond network would prove to be dominant in determining the dynamics of de-excitation, leading to bulk-like dynamics at the water surface.

Below we will first describe results pertaining to the water/air and ice/air interfaces and use these systems as examples to outline what kind of information can be extracted from time-resolved SFG experiment. In the second part, we will then shift to the dynamics of water in contact with different solid minerals.

Below, we will use the publication by Sudera et al.,^43^ which focuses on liquid water and ice in contact with air, as an example (see Figure 2) for the spectral appearance of features in pump–probe experiments and the information which can be inferred from them. This publication not only compares the dynamics of the hydrogen bonded and free OH groups of water in the vibrational stretch region but also explores the impact of structure/phase by comparing liquid water with ice. Since phase transitions between liquid water and crystalline ice commonly take place in the atmosphere, especially in mixed phase clouds, it is important to understand how the phase transition may affect any energy dissipation dynamics in these systems. The homodyne static SFG spectra of both water and ice *I*_h_ in contact with air can be seen in Figure 2.

**Figure 2:**
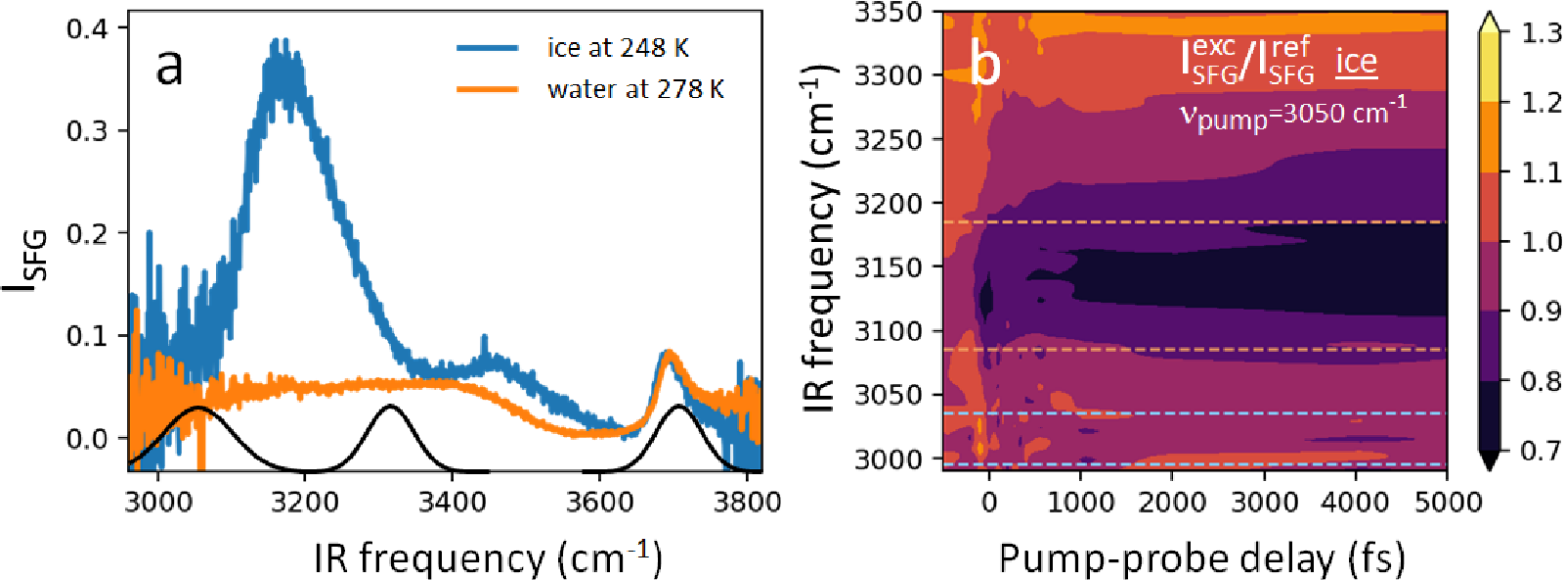
(a) Static SFG spectra of the water/air and ice/air interfaces. The black traces show the spectral components of the pump pulses utilized in this work. (b) Time resolved SFG map of the ice/air interface. The color code indicates the relative fraction of the pumped and unpumped reference spectrum. The former is here referred to as the excited spectrum *I*_SFG_^exc^ and the latter as the reference spectrum *I*_SFG_^ref^. The dashed lines indicate the regions of interest, with the GSB found between the orange boundaries and the ESA signal between the blue boundaries. Reproduced from ref (43). Copyright 2020 Creative Commons Attribution 4.0 International (CC-BY 4.0) license.

The water/air OH stretch spectrum shows two distinct regions: the broad double peak feature of the hydrogen bonded OH groups, extending from 3000 to 3600 cm^–1^ as well as the much sharper free OH peak at around 3700 cm^–1^. The latter corresponds to OH bonds that are not incorporated into the hydrogen bond network and protrude from the liquid bulk into the gaseous phase.

Both regions may provide insight into the water structure (and in the time-resolved experiments which will be discussed later, its excitation dynamics); however, they report on different aspects. The bonded OH band has been described of reflecting more of a collective mode, representative of the delocalized nature of the hydrogen bond network. The free OH however, lacking integration into an intermolecular network, is described as a true local mode and contains very site specific information on the outermost layer of the surface. It is, as such, an excellent probe to study for example the surface coverage of hydrophilic/amphiphilic molecules, as the free OH is strongly affected by increasing surfactant coverage.^44,45^

The SFG spectra of ice and water are good examples to demonstrate this difference: It is clear from the comparison in Figure 2a that while the free OH band is similar in intensity, the bonded OH band of ice is far more pronounced, especially the low wavenumber band at around 3200 cm^–1^. These differences are reflective of the more ordered and rigid hydrogen bond network found in hexagonal ice compared to the disordered liquid phase.

Also displayed in Figure 2a are the spectral components of the pump pulses used in this work to excite the aqueous surfaces: The first sits at the low wavenumber flank of the bound OH, the second centrally at the band, and the third is chosen to excite the free OH band at the high wavenumber side of the OH stretch region.

To maximize interaction between a given vibration and the pump pulse, it is desirable that the polarization of the light and the transition dipole moment of the light are aligned with one another. Since the work presented here mainly focuses on the orientation of water relative to the surface plane, the transition dipole of the relevant vibrations is probed along the *z* axis/surface normal. P polarization of the pump light is therefore preferable when studying these vibrational populations. S polarization may be used when investigating, for example, the asymmetric stretch mode of water or other vibrations with an in-plane transition dipole moment. In the study by Sudera et al., the SFG signal is collected in P-SSP polarization (P-polarized IR Pump; S-polarized SFG; S-polarized Visible; S-polarized IR Probe) across the OH stretch region.

The resulting time-resolved spectra are usually displayed as a map of the SFG intensity of the pumped spectrum in relation to its unpumped counterpart, with the vibrational frequency and the pump/probe delay spanning the two respective axes, as can be seen in Figure 2b.

In general, the impact of the IR pump on the SFG spectrum can be described in terms of two main spectral components: Ground state (GS) bleach and excited state (ES) signal. The ground state bleach (GSB) corresponds to a reduction of ground state SFG signal since a part of the population has been lifted into the excited state, and the ESA signal is observed as an additional feature due to SFG emission from the vibrationally excited state (see Figure 1). In the example shown here in Figure 2b the pumped spectra are divided by their unpumped equivalent () to yield the displayed map. The ground state bleach is noticeable as the dark shaded area with a relative SFG intensity below 1. The generated excited state (ES) population in turn gives rise to an additional excited state SFG band in the pumped spectrum (which is shown in Figure 2 as a contribution >1, shaded in orange and yellow). Due to the anharmonicity of the system the ES signal appears spectrally shifted to lower frequencies. Both the GSB and ESA features appear in the time-resolved map after *t* = 0 and develop as the system subsequently relaxes over time and the excited population returns to the ground state.

To better trace the dynamics of these spectral contributions over time, the SFG signal is often integrated across a given spectral region, as for example between the orange and blue dashed lines, respectively, in Figure 2b. The integrated intensity can then be displayed as a function of time, as shown in Figure 3. Here the impact of both the GSB and ESA become much more apparent as the relative SFG intensity either dips or peaks relative to the unpumped case, i.e., a value of 1.

**Figure 3:**
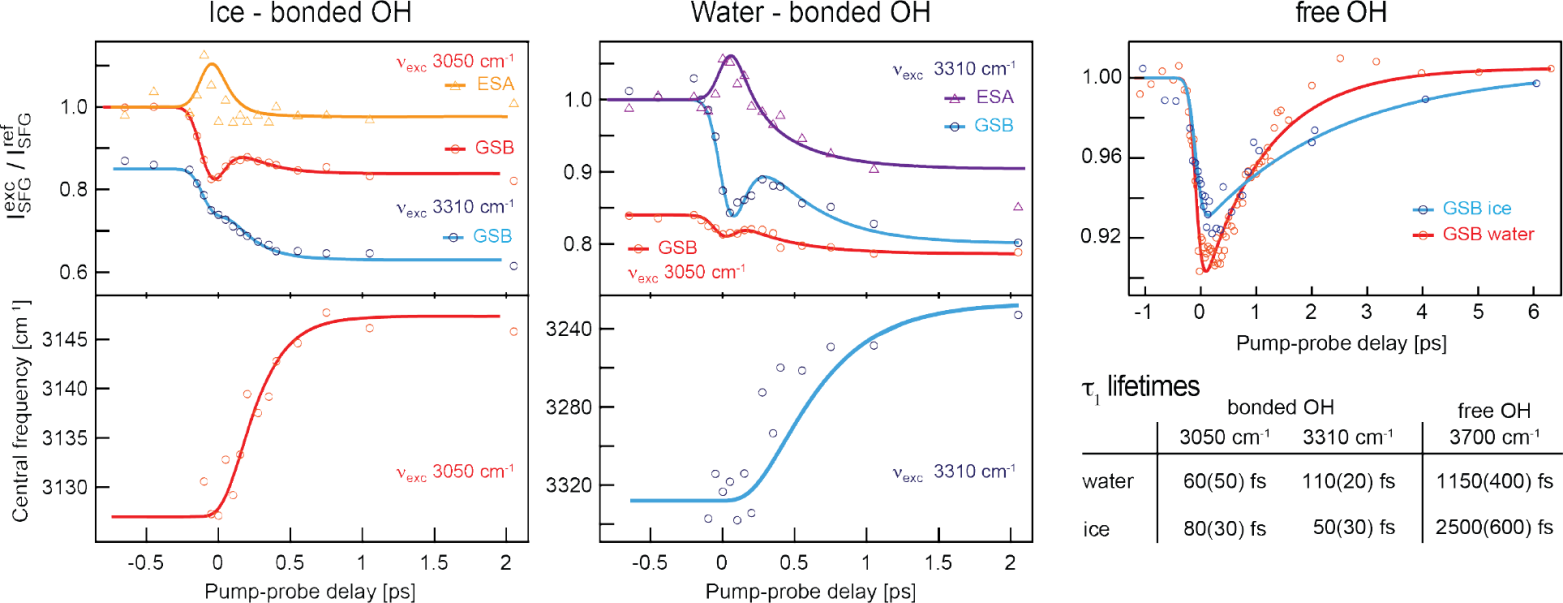
Dynamic traces of water and ice surfaces and the centerline frequency as a function of time for both the bonded OH and free OH region, as well as their corresponding τ_1_ lifetimes. Triangles show ESA and spheres show GSB. The red and orange traces are pumped at 3050 cm^–1^ and the blue and purple traces are pumped at 3310 cm^–1^. The solid lines are based on the respective models used to describe the bonded and free OH data sets: in the case of the bonded OH regions, a double exponential decay derived from the 4-level-model (see Figure 1) is used, and a single exponential model is used for the free OH data. The insert in the bottom right summarizes the relevant τ_1_ lifetimes extracted from the data. Figure based on data previously published by Sudera et al.^43^

In Figure 3 it also becomes apparent that a single exponential is insufficient in describing the GSB dynamics of the hydrogen-bonded OH stretch dynamics, similar to the relaxation dynamics observed in bulk water.^46^ Instead a double exponential—derived from the 4-level model^47,48^—has to be used. Here the overall process, as depicted in the inset in Figure 1, is described in terms of first the vibrational excitation of, for example, the OH stretch, lifting a population of molecules from their vibrational ground-state ν_0_ into the first excited state ν_1_. This state has a given lifetime τ_1_ which depends on both the nature of the excited state and the chemical environment of the molecule. The first step of the relaxation process is usually a transition into a secondary excited state ν*. In the case of water this is most likely an overtone of the bend mode, which is well matched with the stretch in terms of frequency. From there the system may then relax further via internal conversion until eventually all excess energy is dissipated in the form of heat. The final state the system reaches on the picosecond time scale is therefore a heated ground state ν_0_^*^. Due to the excess thermal energy the overall hydrogen-bonding strength is weakened in the heated ground-state, leading to a shift in the observed SFG signal,^49^ as can be seen in Figure 3. Since the dissipation of the excess thermal energy happens on a longer time scale, the heated ground state is considerably long-lived and the true ground-state ν_0_ is often not observed within the range of pump/probe experiments.

In Figure 3, the results of this model are presented as solid lines, and the obtained τ_1_ lifetimes are summarized in the inset. The mechanism through which this OH population dissipates the excess energy at the surface has been discussed extensively in the literature previous to the publication by Sudera et al. In the case of the bonded OH stretch, time-resolved experiments on the air/water interface^46,50,51^ showed that the relaxation dynamics were again highly reminiscent of bulk water.

To investigate the difference in relaxation dynamics between the bulk and the surface of water, van der Post et al.^46^ compared vibrational lifetimes obtained by bulk pump/probe IR spectroscopy with time-resolved HD SFG, the latter employing a P-SSP polarization scheme. Specifically, the authors found that for both bulk and surface water the vibrational relaxation time changes across the bonded OH band. For bulk water it increases from 250 fs (at 3100 cm^–1^) to 550 fs (at 3700 cm^–1^). Water at the surface shows a similar behavior with the relaxation time increasing from 150 fs (at 3100 cm^–1^) to 750 fs (3500 cm^–1^), however at different absolute values compared with bulk water. This observation can be quantitatively described by a model including spectral diffusion and intermolecular vibrational energy transfer to the overtone of the bend.

A unique structural aspect of the water interface is that its geometry necessitates that the hydrogen bond network be frustrated, leading to the formation of free OH bonds, which protrude from the liquid phase into the gas. Spectrally these OH groups may be observed as a separate band in the OH stretch region. The high vibrational frequency of around 3700 cm^–1^ and narrow bandwidth of this feature reflects the lack of a stabilizing hydrogen bond in this OH population. Interestingly, the vibrational dynamics of the free OH modes is also markedly different from that of the bonded OH, since the vibrational de-excitation can be described by a single exponential (see Figure 3 —the single exponential model fitted to the experimental data is displayed by a solid line). This indicates that the ground state of the system is recovered without the involvement of an intermediary state, as described in.^52,53^ The main pathway for relaxation of the free OH population at the water surface proceeds via diffusive reorientation of the surface molecules. This reorientation leads to the excited free OH bond becoming integrated into the hydrogen bond network, thereby being transformed into an excited bonded OH. At the surface the OH group of a different water molecule may be liberated and replenish the population of free OH groups in the vibrational ground state.^52,53^ The excess vibrational energy is then dissipated via the hydrogen bond network. The lifetimes obtained for the free OH vibration at the water and ice interfaces are summarized in the inset in Figure 3.

This mechanism can also rationalize why the single-exponential model is sufficient in describing the relaxation dynamics: The excess energy of the free OH excitation actually moves into the hydrogen bond network through reorientation (not intermolecular coupling), where it subsequently follows the pathway of dissipation to a secondary state and finally into the ground state (as previously outlined for bonded OH excitation). However, these dynamics would then be noticeable in the bonded OH region. The dynamics observed in the free OH region therefore reflect the diffusive exchange of excited free OH groups for free OH groups in the vibrational ground state rather than intramolecular vibrational relaxation. The relevant time constant of relaxation is, therefore, determined by the rate of molecular reorientation at the surface. In the case of the free OH, the limiting step for the dissipation of energy is, therefore, molecular reorientation rather than the coupling between vibrational modes or with other molecules.

In terms of the comparison between the two condensed phases of water, Sudera et al.^43^ found that the vibrational relaxation of the bonded OH groups is accelerated in ice compared with liquid water (see Figure 3 and table therein), whereas those of the free OH group are slowed down (see Figure 3 and table therein). In the case of the bonded OH groups, this can be rationalized as reflective of the increased strength of the hydrogen bond in ice which enhances the intermolecular coupling between the individual water molecules. Considering the reduced dynamics of the free OH two possible explanations are presented: (1) The reorientation of the water molecules containing a free OH group might be hindered by the more rigid surface structure of ice, thereby reducing the transformation of the free OH into a bonded OH group, (2) intramolecular energy transfer, which is the second possible pathway for relaxation, is likely also slower in ice due to the larger frequency mismatch between the free and the bound OH. Considering the previously discussed work by Inoue et al.^53^—showing that diffusive reorientation is the dominant pathway for relaxation of the free OH groups—the reduced dynamics of the free OH groups in ice is therefore likely due to the rigidity of the crystalline network. At lower temperatures, the mobility of water groups at the crystalline surface is further reduced, and therefore it takes longer for the excited free OH groups to reorient and reintegrate into the hydrogen-bond network.

In summary, the more rigid structure of ice and the stronger intermolecular hydrogen-bond network appear to accelerate the energy dissipation along the hydrogen-bond network, leading to a decreased vibrational lifetime in the bonded OH region. The same structural rigidity of ice may also lead to the excitation in the free OH mode being more long-lived and energy becoming kinetically trapped at the surface. Thus, it becomes clear that the vibrational relaxation dynamics in the bonded OH region are reminiscent of those of the bulk liquid, which is likely due to the high resilience of the hydrogen bond network. While there is almost no impact of the truncation of the hydrogen-bond network, it is clear that the phase and changes in the structure will have an impact on the dynamic processes occurring at the interface.

It is well described in the literature^5^ that many solutes and surfactants change the structure of the hydrogen-bond network close to the surface. Based on the results presented so far, it is therefore reasonable to assume that the manifold of solutes found in atmospheric aerosol might also have an impact on the energy dissipation dynamics across the hydrogen bond network.

For example, Deiseroth et al.^54^ have studied the influence of common ions found in atmospheric aerosols (Na_2_CO_3_ and Na_2_SO_4_) on the vibrational energy dissipation at the water surface. The authors compare the SFG spectra, obtained in SSP polarization, of the saline solutions with pure D_2_O, which all feature the same bands, namely the broad double peak bonded OD band around 2200–2600 cm^–1^ and the more narrow free OD peak at around 2750 cm^–1^. In this study, D_2_O was chosen over H_2_O; however any insight on the impact of ions on the observed dynamics are expected to also hold for the equivalent H_2_O system. The studied ions are found to clearly influence the water structure, as observed via changes in the shape and intensity of the bonded OD band in both homodyne and heterodyne SFG spectra (Figure 4a, b).

**Figure 4:**
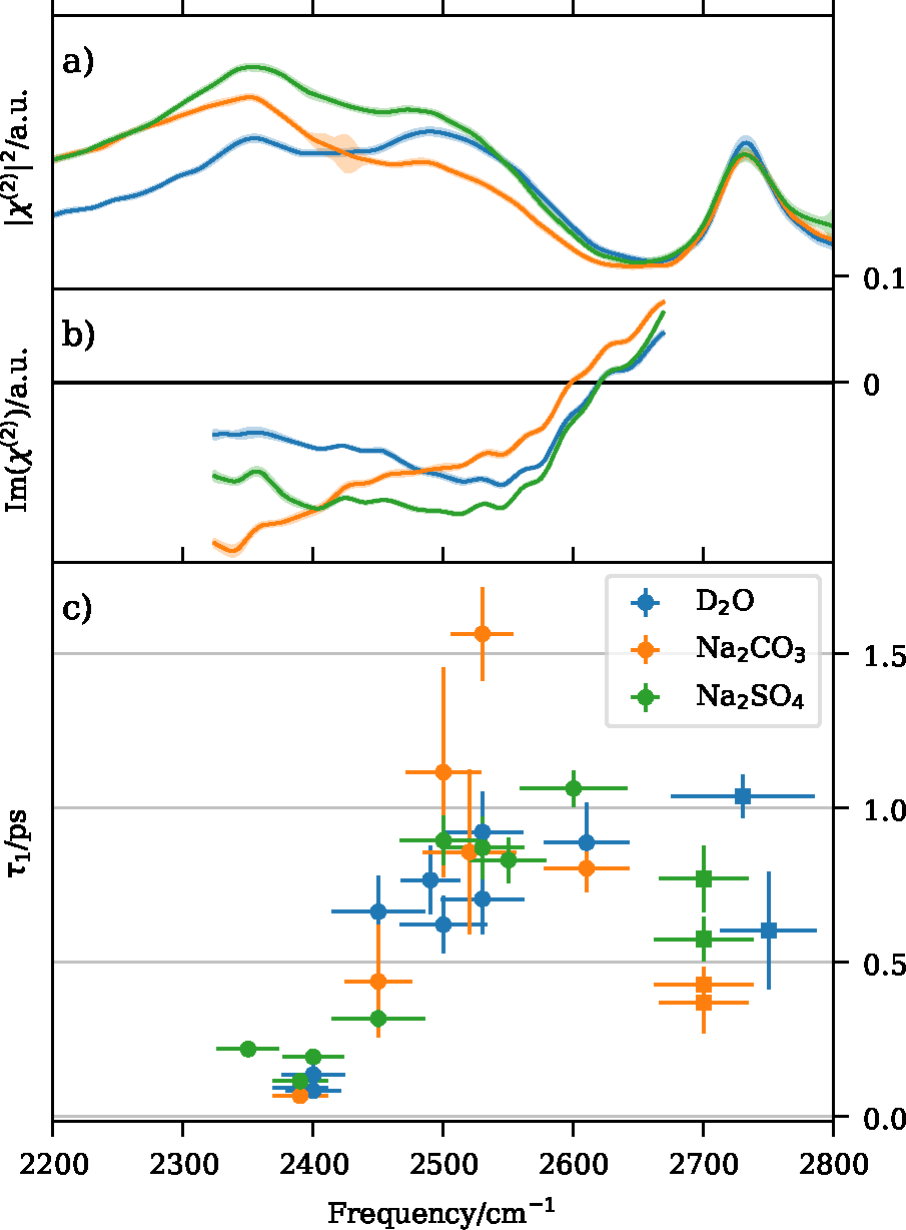
Homodyne (a) and heterodyne (b) SFG spectra of D_2_O (blue) as well as Na_2_CO_3_ (orange) and Na_2_SO_4_ (green) solutions. Panel (c) shows the vibrational lifetime τ_1_ across the OD stretch band of these systems. While it is apparent that the presence of these salts at the surface affects the structure of the interface, see the changes in intensity and shape of the static SFG signal in (a) and (b), the lifetime of the vibrational OD stretch mode remains unaffected within the accuracy of these measurements. Reproduced from ref (54). Copyright 2019 Creative Commons Attribution International (CC-BY) license.

Specifically, in the presence of Na_2_SO_4_ the intensity of the low wavenumber flank of the bonded OD band is increased compared with that of water. This is traced back to a corresponding increase in the intensity of the negative band in the imaginary part of χ^(2)^, as observed by HD-SFG (Figure 4b). When the system contains Na_2_CO_3_, the homodyne spectrum of the bonded OH band is also more intense on the low wavenumber side compared with water, but the intensity is decreased on the higher wavenumber side (Figure 4a), with the corresponding behavior being observed in the heterodyne spectrum (Figure 4b).

In the studied systems, the relaxation dynamics after excitation in the OD stretch region using multiple different IR frequencies in a P-SSP polarization scheme (as observed via τ_1_ in the 4-level model) remained unchanged across the saline systems and compared with water (see Figure 4c). This clearly indicates that the heat dissipation pathways at the water/air interface are remarkably robust to structural changes that may occur in the hydrogen bonding network due to ionic solutes. However, this might be related to the fact that these ions do not show a pronounced surface enrichment. Their impact on structure and orientation in the surface region may therefore be small enough such that the surrounding hydrogen bond network can compensate for the resulting changes in terms of the observed vibrational relaxation.

The first time-resolved SFG experiments by McGuire and Shen in 2006 were performed on solid/liquid interfaces with the aim to study how the hydrogen bond network of water reacts to the truncation at the interface.^42^ The authors used an IR pump of variable frequency and P polarization, leading to a P-SSP polarization combination.

In order to separately study the dynamics of free and bonded OH groups at the water surface, the authors investigated water in contact with a hydrophilic silica and hydrophobic OTS (octadecyltrichlorosilane) coated silica surface. In the case of the free OH groups a single-exponential model was used to describe the dynamic behavior. In the case of the bound OH the familiar 4-level/double-exponential model used for bulk water was found to describe the dynamics occurring at the surface well. As previously outlined this model assumes an initial vibrational relaxation and a subsequent thermalization of the system (see inset in Figure 1). The time scales determined using this model were found to be remarkably similar to those in the bulk, indicating that the structural differences between the hydration geometries in the bulk and at the interface do not strongly influence the dynamics of the hydrogenbond network at ultrafast time scales. This in turn suggests that the hydrogen bond network is rather resilient and can restructure to accommodate the loss of hydrogen bond partners at the interface.

Another important aspect of solid/liquid interfaces is that mineral surfaces may acquire a surface charge when they are in contact with aqueous solutions of different pH. The sign and magnitude of the surface charge depends both on the pH of the solution and the nature of the mineral. Depending on the composition of the surface the point-of-zero-charge (PZC) may be found at different pH (see for example Figure 5), with the surface acquiring charge of opposite sign at pH above and below the PZC.

**Figure 5:**
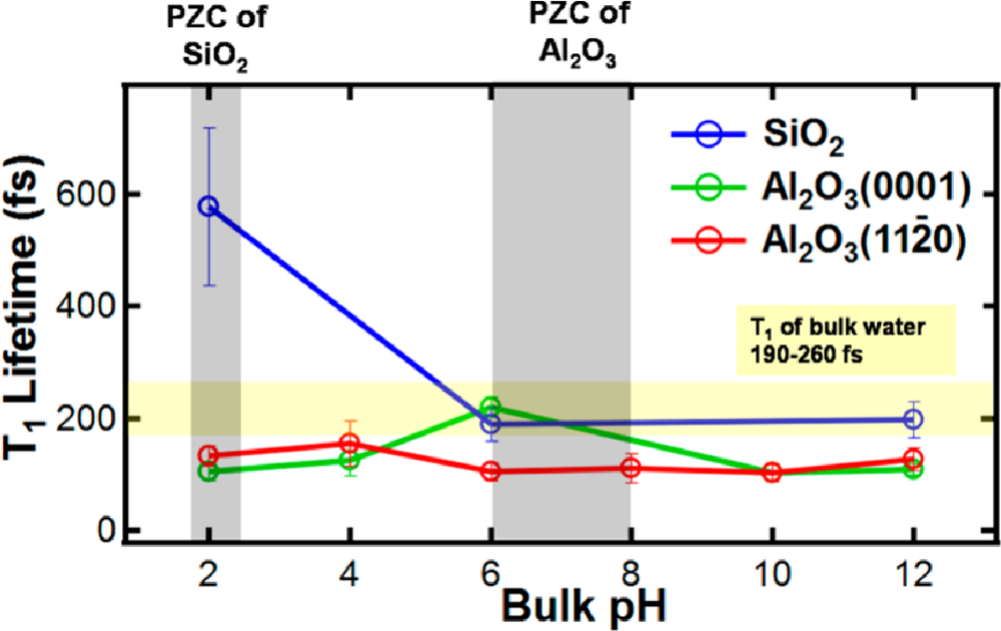
Effect of the surface charge at different pH values of the subphase on the vibrational lifetime of surface OH groups at different mineral interfaces. The gray areas indicate the point of zero charge of SiO_2_ and Al_2_O_3_ respectively. The yellow region represents the range of values reported for the vibrational lifetime of bulk water. Reprinted from ref (57). Copyright 2017 American Chemical Society.

The impact of this pH-dependent surface charge on the vibrational dynamics of water has been explored by Eftekhari-Bafrooei et al. in the case of the fused-silica interface^55^ using time-resolved measurements in P-SSP polarization. The PZC of silica is found at about pH 2, as shown in Figure 5.^55^ Since the solid surface may acquire a different charge depending on the pH of the liquid subphase, it might be reasonable to assume that the vibrational relaxation dynamics are also affected by the resulting surface potential. The authors therefore compared the vibrational dynamics of water across a range of pH conditions at the water/silica interface. It was found that at high pH, and subsequently high surface charge, the relaxation dynamics of the OH stretch closely resemble the fast dynamics observed in bulk water. However, at the point of zero charge of the silicon/water interface, the vibrational lifetime more than doubles (see Figure 5). This indicates that the excited state is significantly less long-lived at higher pH values (and surface charge) of the liquid solution. This effect may be related to the degree of structuring that is imposed by the charge induced external field and the resulting intermolecular coupling.

To further study the relaxation dynamics of the OH stretch in contact with silica, Eftekhari-Bafrooei et al.^56^ performed time-resolved SFG experiments (using P-PPP polarized light) of isotopically diluted water (i.e., HOD in D_2_O) in contact with silica at different pH, focusing on changes in lifetime depending on the frequency of the IR pump pulse. In this study the authors find a dependence of the vibrational relaxation on the frequency of the pump across the bonded OH stretch band, namely, that it is shorter on the low frequency (red) side of the OH stretch band and longer at the high frequency (blue) side at both pH 2 and pH 12.^56^ However, lifetimes at pH 12 are shorter compared with pH 2 at the studied pump frequencies, as outlined in the previous paragraph^55^ for pure H_2_O. However, unlike in H_2_O/silica interfaces, the authors find that there is a shift in the static HOD/silica spectra depending on pH and therefore surface charge. Specifically, the bonded OH band red-shifts at higher pH and surface potential. The authors correlate this to an increased order in the hydrogen bond network and stronger intermolecular interactions due to the added electrostatic field. The faster relaxation dynamics at pH 12 is therefore attributed to stronger hydrogen bonding in the surface region. The change in lifetime across the bonded OH band might be attributed to two different effects: either the changing hydrogen bond strength contributing to the SFG signal across the band (with the stretch mode of another molecule being the main accepting mode) or the energetic proximity to the HOD bend mode (with the bend mode being the main accepting mode). However, the data in this work do not allow for differentiation between these two mechanisms.

The same research group subsequently studied the Alumina(0001)/water interface^57^ using P-PPP time-resolved SFG experiments. In the case of Alumina the PZC is expected to be found between pH 6 and 8, as shown in Figure 5. The authors found that the vibrational dynamics of the bonded water OH stretch is accelerated at charged Al_2_O_3_ surfaces compared with the equivalent relaxation process in bulk water and at charged SiO_2_ surfaces (see Figure 5). This change in relaxation dynamics could be due to either fast proton transfer dominating the vibrational relaxation and/or efficient coupling between the OH stretch and the bend overtone via the presence of low frequency (approximately 3000 cm^–1^) OH stretching modes. Lastly, the addition of excess ions (0.1 M NaCl) seems to have little to no effect on the time scale of the observed vibrational dynamics, which is in contrast with the behavior observed at the silica surface.

The influence of ions on the solid/liquid interface has been of interest over the years, since they not only screen any surface charge that may be present but are also known to perturb the hydrogen bond network in the bulk. In the case of the Alumina(0001)/Water interface^58^ Tuladhar et al. studied the changes that occur not only to the hydrogen bond network using static SFG in PPP polarization but also to the resulting vibrational dynamics under a variety of conditions, using an additional P polarized pump beam. The solid alumina was brought in contact with both alkaline and acidic solutions (pH 4/10) containing different sodium halide salts (NaF, NaCl, NaBr, and NaI), which allows us to not only draw conclusions about the general presence of ions but also determine if there is a chemically specific impact of the halides on the observed dynamics.

In the case of positively charged alumina surfaces, i.e., pH 4, the halide ions are expected to directly act as counterions screening the surface charge. The closer these halide ions are to the surface, the better their screening ability and hence the smaller the SFG signal is expected to be. The halide ions are observed to attenuated the SFG signal in the sequence F^–^ ≫ Br^–^ > Cl^–^ > I^–^. This indicates that F^–^ has the largest affinity for the alumina interface and I^–^ the smallest, with Br^–^ and Cl^–^ displaying intermediate affinities. However, the latter two do not follow the Hofmeister series entirely, which the authors attribute to inconsistencies in ion affinity toward the alumina surface.^59,60^ The authors suggest that the uniquely large screening effect of F^–^ is possibly due to the ion adsorbing onto the surface, thereby displacing water molecules and severely disrupting the hydrogen bonding structure at the surface.

In terms of the vibrational dynamics of water at these interfaces it was found that only fluoride perturbs the vibrational lifetime of water next to a positively charged alumina surface, slowing it down by a factor of 4.^58^ This effect is again attributed to the fluoride ion’s larger surface affinity, leading to the disruption of the strong hydrogen bonding interaction between the surface Al–OH groups and the nearby water molecules. The other larger ions do not affect the studied dynamics, which indicates that the water hydrogen network may compensate for the disturbance caused by the presence of nonabsorbed counterions in terms of the vibrational relaxation dynamics.

In the case of the negatively charged alumina surface at pH 10 the main counterion responsible for shielding the negatively charged alumina/water interface is Na^+^, which is common to all solutions. However, anion specificity is still observed at negatively charged alumina surfaces. The presence of the specific anions again leads to attenuation of the SFG signal. The authors suggest that this might be due to the Na^+^ ion and its respective counterion adsorbing to the negatively charged surface as an ion pair. The sequence of attenuation was found to be Br^–^ > I^–^ ≃ Cl^–^ > *F*^–^ following the inverted Hofmeister series, apart from I^–^. The authors speculate that the most likely explanation for this sequence is a slightly higher affinity of the larger ions toward the negative surface, allowing for more Na^+^-Br^–^ or Na^+^-I^–^ ion pairs to adsorb. The determination of the surface affinity of these negative ions is unfortunately not straightforward in the case of a negative interface, and the precise mechanism of these screening effects is therefore still speculative.

When investigating the relaxation dynamics of water at negatively charged alumina surfaces the authors find that the dynamics are distinctly different from those of the positively charged surface. Specifically, the data can no longer be fully described by the 4-layer model, as the dynamics during the initial 100 fs are limited by the instrument response function of this specific experiment. The origin of this fast component is unclear to the authors, however it appears to be sensitive to both the pH and the nature of the halide ion (NaBr > NaI > NaCl > NaF, following the same sequence as for the attenuation of the static SFG described above), making it unlikely to be an experimental artifact. Using the techniques outlined in the original publication and in this perspective, it is currently unlikely that the origin of this component can be further explored, mainly due to the inherent limitation by the temporal laser-pulse profile. The mentioned correlation with the static SFG intensity might suggest that it is in some manner associated with the hydrogen bond environment at the surface.

Neglecting the fast components, the authors determine a lifetime for the vibrational dynamics of water using the 4-level model and find that the τ_1_ component remains unaffected by the nature of the halide ion. This suggests that in this case there is no direct link between the attenuation of the static signal and the vibrational lifetime.

Different alumina surfaces were also studied, such as the Alumina(112̅0)/Water surface.^61^ In this study static SFG spectra were acquired in both SSP and PPP polarization; however, time-resolved experiments were performed in P-PPP polarization. In this case the authors found no pronounced variation of the relaxation dynamics across the studied pH range (see Figure 5). Additionally the static SFG spectra showed a pronounced OH stretch peak at 3000 cm^–1^, which was also persistent in the presence of hydrated ions. This band was assigned to either chemisorbed OH groups on the surface or interfacial water molecules that form strong hydrogen bonds with the aluminol groups at the surface. The authors suggest that the presence of this population of very strongly hydrogen bonded OH groups might be the reason for the fast and invariant relaxation dynamics observed across all pH dependent measurements. These experiments also highlight that the nature of the exposed surface may lead to very different relaxation dynamics, even when the same mineral is studied.

The impact of surface charge was also investigated in the case of D_2_O in contact with CaF_2_ surfaces by Lesnicki et al.,^62^ who studied the intermolecular vibrational energy transfer of water at low pH using P-SSP time-resolved SFG experiments. In the case of CaF_2_ the low pH leads to the partial dissolution of fluorite ions from the surface, and in turn a very highly positively charged mineral surface is formed. The vibrational lifetime of the OD stretch mode was found to be comparable to that of bulk water, but substantially faster than that of the water/air interface^63^ and even other charged interfaces, such as water/lipid interfaces.^64^ However, when the lifetimes after excitation were compared across the range of the OD band, two distinct regimes were observed. At higher wavenumbers, above 2500 cm^–1^ the vibrational lifetime was found to increase with the frequency of the pump pulse, whereas at lower and intermediate frequencies across the OD band the lifetime appeared to plateau. In bulk water, the heterogeneity across the OH/OD band was explained by the frequency dependent coupling to the overtone of the bend mode. This effect was also related to the observed heterogeneity of the high frequency regime in this work. To explain the plateau at low and intermediate OD stretch frequencies, the authors employed molecular dynamics studies to further investigate the geometry of water molecules at the interface. The authors find that in these simulations, some water molecules become pinned close to surface defects consisting of positively charged fluoride holes and have a unique hydrogen bond environment. Specifically, they only donate hydrogen bonds and do not accept any. Similarly the surrounding interfacial water molecules also experience a high degree of order and an incomplete solvation shell but to a lesser degree. These effects were found to contribute to a more stable and ordered hydrogen bonding network at the interface, which in turn leads to the fast relaxation dynamics observed below 2500 cm^–1^. These results highlight how a relatively small population of charged surface sites can have a pronounced impact on the local hydrogen bond geometry and, thus, the vibrational dynamics of these systems.

The work discussed in the previous section makes significant progress in understanding how the system may dissipate excess vibrational energy. However, many mainly organic molecules found in atmospheric aerosol particles can also absorb light in the visible part of the solar spectrum (most likely inducing a HOMO/LUMO transition) without triggering a subsequent dissociation or other reaction. This absorption of a visible photon, and the associated electronic excitation, can lead to a substantial redistribution of electron density, which will subsequently induce a reorientation of the molecules solvation shell.

Due to the high fluence of sunlight in the atmosphere, it is highly probable that at any point in time a significant subset of molecules is excited, and considering only their ground state orientation/hydration relative to the surface may be insufficient in describing their hydration structure and possibly even reactivity.

Questions about how quickly the solvation shell may relax following such a change in the electron distribution can determine, for example, the lifetime of transition states or perhaps even how quickly the solvation environment can optimize its structure to hydrate newly formed species along different reaction pathways.

These questions have been addressed by Rao et al.,^65^ who studied the solvation shell dynamics of surface active coumarin 314 using SSP polarized SFG in response to a change in the local electron density distribution. The latter was induced by the absorption of a p-polarized 423 nm photon preceding the SFG probe pair, corresponding to the S_0_ to S_1_ transition. Since the first excited state is nondissociative, the atomic lattice of the molecule remains largely unaffected and the solvation shell relaxes according to the changed electronic structure, specifically the increased dipole of the molecule from approximately 8 to 12 D.

In the case of coumarin 314 the observed dynamics were similar to that of coumarin 242 (also a coumarin compound that has a similar structure) in bulk water, which indicates that the properties of the first solvation shell largely stay the same. However, it remained unclear in this study what the influence of the friction the first solvation shell experiences with the surrounding water network during rearrangement is.

Another interesting approach to study the structural rigidity of the surface is to displace the solute itself. In a subsequent work, Rao et al.^66^ have used circularly polarized visible light to study the rotational relaxation dynamics of coumarine 153 relative to the surface normal. Using circularly polarized light incident along the surface normal allows for a uniform excitation of molecules along the in-plane axis, as opposed to linearly polarized light, and therefore the selective study of out-of-plane rotational relaxation dynamics.^67^ The relaxation of the molecule after excitation is probed via polarization dependent SFG, employing SSP and SPS polarization, to monitor the recovery of the ground state orientation.

The static SFG response of coumarine 153 in its ground-state was initially studied in all four unique polarization combinations, namely, SSP, PSS, SPS, and PPP. Subsequently, the effect of the circularly polarized pump beam was observed in SSP and SPS polarization. In this study the combination of different polarizations is used to deduce the average orientational angle of different parts of the coumarine molecule. The dynamic effects induced by this pump pulse are ultrafast solvation dynamics, orientational dynamics, and eventually population recovery, all of which are well separated in time. An example of solvation dynamics has already been discussed above, and similar approaches were used in the literature employing SHG spectroscopy.^68,69^

The orientational displacement following the visible pump pulse is studied via the relative SSP and SPS SFG intensity of two marker groups within the molecule, namely, the −C=O and −CF_3_ groups. These traces are then used to determine the time dependent average orientation of the two groups and therefore the angle of the molecule normal (which was defined to be perpendicular to the plane of the benzene ring) relative to the surface normal. The molecule normal was found to return to its ground-state orientation with a time constant of 126 ps. Remarkably, the orientational diffusion coefficients of the two marker groups are 2 orders of magnitude slower than those found in bulk. The authors also show that the molecule experiences much more pronounced out-of-plane friction compared with the bulk, indicating a highly ordered and rigid surface structure. This work is contextualised within the “wobbling-in-a-cone” model which has often been used to describe the orientational flexibility of molecules at the surface.^70,71^ More detailed knowledge about the properties of this cone can help in understanding the variety of surface structures as well as the range in which a surfactant is able to reorient in a barrier-free manner. The latter is especially important when it comes to, for example, interaction with a reactant and the point at which this reorientation becomes hindered by the solvation environment.

This approach can facilitate the study of the orientation/rotation potential the molecule experiences at the water surface and more accurately determine the degree of order and the flexibility of a surface film, both of which are important in understanding not only the ground state dynamics but also how quickly reaction products are able to reorient themselves on a water surface.

In many photoactive molecules, the absorption of light with sufficiently high energy leads to a photochemical dissociation reaction. This of course requires that the molecule can not only be electronically excited (as discussed above) but that the excited state which is reached after light absorption is fully dissociative or that a dissociative state is subsequently populated from the state into which the molecule is excited (via, for example, a conical intersection). While many molecules can display such photochemical reactions, as is manifest in the vast literature that exists on bulk photochemistry, only a select few have been studied at the air water interface. Since the unique solvation environment can lead to changes in the ground and excited state energies, it is unclear whether the reaction rates determined for bulk reactions still hold relevance when considering surface reactions. If anything, it would be reasonable to assume that rates would be largely different.

Two particularly interesting works that make use of time-resolved SFG to study such photochemical dissociations are discussed below, both of which highlight the role the interface plays in the dynamics of these reactions.

One of the simplest photochemical reactions is the transfer of an electron to the solvent, by either a solvent or a solute molecule. Matsuyaki et al.^72^ have studied both cases to assign the transient spectroscopic signature of a partially hydrated electron generated at the water surface. This was achieved by either two-photon absorption (in the case of the parent molecule being water) or single-photon absorption of the indole. The molecular response to the p-polarized UV pulse beam was monitored by time-resolved HD-SFG in SSP polarization. The lifetime of the solvated electron at the surface was found to be 100 ps, after which it appears to dissolve into the bulk to become fully hydrated rather than recombine with its parent ion. Additionally, the wavenumber of the transient band was determined to be 3260 cm^–1^ and corresponds to the OH stretch vibrations of the water molecules which are directly interacting with the electron in its hydration shell.

The possibility of the surface affecting the energetic landscape of different reactions has been discussed before,^39,40^ but a recent publication by Kusaka et al.^73^ clearly demonstrates the profound impact the interface can have via the case of UV absorption induced phenol dissociation. This reaction takes place by producing a phenoxy radical as well as a hydrated electron and proton (see Figure 6). This study again employed time-resolved UV pump HD-SFG probe spectroscopy using a P-SSP polarization scheme.

**Figure 6:**
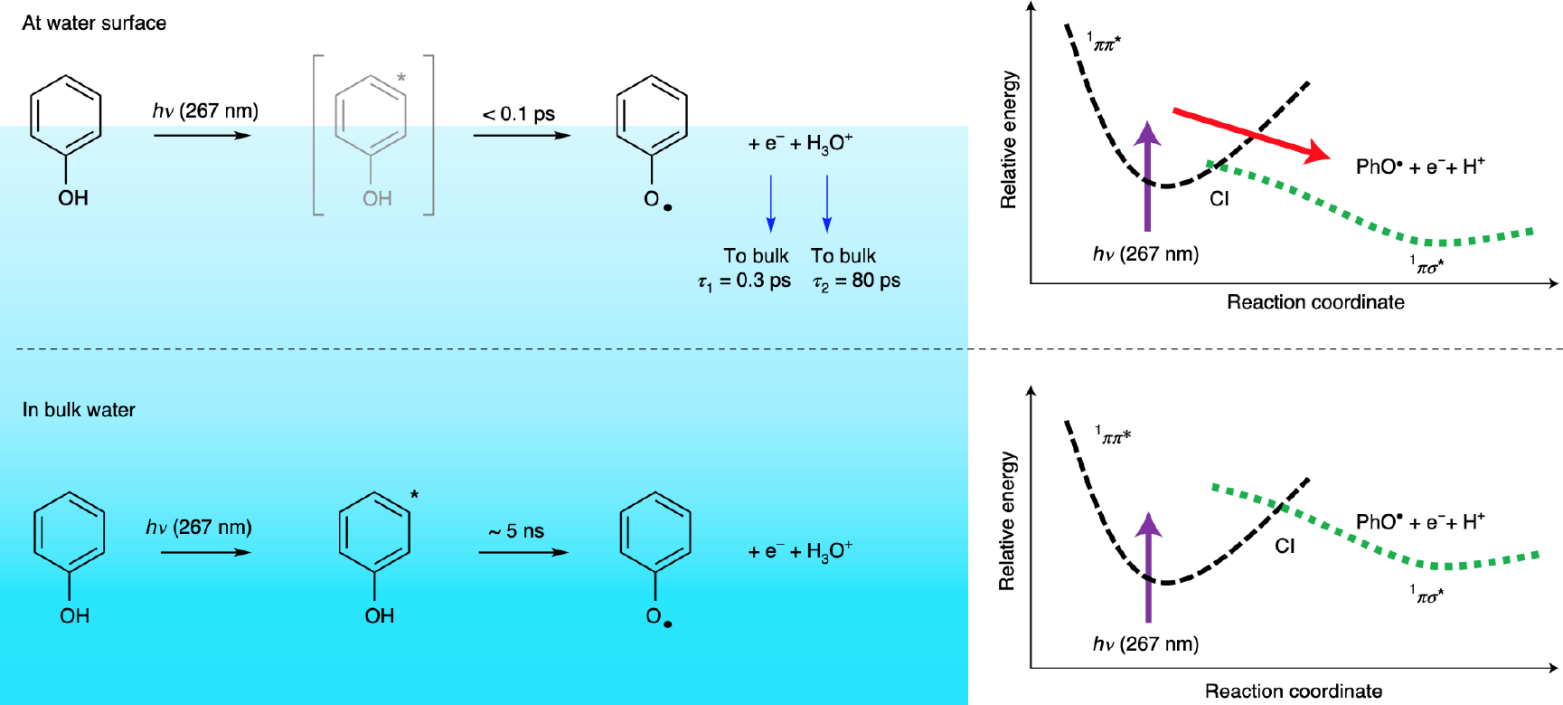
Photochemical dynamics of phenol at the water surface (top) and in the bulk (bottom). The sketch on the left-hand side indicates the relevant steps and associated time scales of the reaction, and the diagram on the right-hand side shows the potential energy surfaces of the excited state and the conical intersection (CI) to the dissociative final state. The latter is shifted at the surface allowing the reaction to proceed much faster at lower pump energy. Reprinted from ref (73). Copyright 2021 Springer Nature.

The dynamics of this reaction in bulk have been studied in detail previously,^74^ and the kinetics of the light induced phenoxy radical generation (i.e., the second step of the reaction above) have been found to proceed relatively slowly on the order of around 5 ns, after 267 nm light exposure.

However, when the same reaction was studied at the interface (note: phenol is surface enriched), it was observed that the reaction becomes ultrafast and proceeds in less than 0.1 ps (see Figure 6), making it 10^4^ times faster than in the bulk.

The progression of the reaction was observed via three different transients that appear in the time-resolved HD-SFG spectra, namely, the hydrated electron, the H_3_O^+^ ion, and the phenoxy radical. Both the hydrated electron and the phenoxy radical spectrally manifest indirectly via their interaction with the OH groups that are in direct interaction with the two species (that is their hydration shell). The authors also did not observe any indication of recombination at the surface. Both the hydrated electron and the hydronium ion diffuse into the bulk.

Interestingly, the authors discuss that the same reaction can also become ultrafast in the bulk, but only when the wavelength of the exciting UV light is reduced to 200 nm,^74^ which is just above the conical intersection of the first two electronically excited states. They therefore conclude that the interfacial geometry (and hydration environment) dramatically lowers the conical intersection, thereby allowing the reaction to proceed through its ultrafast pathway at lower excitation energies.

The energetic reasons for this lowering of the conical intersection were later explored by Ishiyama et al.^75^ using ab initio techniques. The authors found that the energetics and position of the conical intersection are dependent on the hydration environment of the phenol molecule. The inherent asymmetry of the hydration shell at the interface thus lowers the dissociative excited state relative to the electronic ground state. The position of the conical intersection is therefore reduced in kind, reducing the energetic barrier to move into the dissociative final state and speeding up the reaction, as sketched in Figure 6.

Since photochemical reactions are always governed by the energetic landscape of the photosensitive molecule, which is in turn affected by the hydration environment, it stands to reason that the shifting of electronic states and their intersections is likely a general phenomenon that may take place on the interface. This therefore makes it challenging to extrapolate the dynamic properties a molecule displays in the bulk to the interfacial geometry and calls for a more detailed study of these processes not only at the water/air interface but also at solid/liquid interfaces.

In order to expand the current research toward more realistic systems, a direct continuation of the work investigating the impact of ionic solutes on the relaxation dynamics at air/water and mineral/water interfaces would be to extend this line of research to include other atmospherically relevant ions, as well as larger organic surfactants which are commonly found in atmospheric systems.

A clear extension of the work discussed above would be to follow reactions which go beyond simple photodissociations. The works in the current literature do not include any subsequent radical reactions, which are, however, very common in atmospheric particles. The current understanding is therefore limited to the initial step of the radical reaction with the smaller reaction products leaving the surface entirely. Since this first step has already proven that the energetic landscape of the surface can vary quite strongly from that of the bulk, it would be an interesting line of research to explore if this also extends to any subsequent reactions. The study of such complex pathways is undoubtedly challenging, since one would have to disentangle contributions of the desired reaction pathway from other changes in the system such as solvent heating and subsequent thermalization as well as experimental instabilities. However, a careful choice of reactants as well as support from theoretical work may support such efforts. Newly formed species may be observed in different spectral regions, well separated from the solvent bands, depending on their respective molecular transitions, making such work at least feasible in the future.

As mentioned in the introduction, the flat surface has so far been used as a model system for the aerosol phase, which is a good assumption for large particles. However, the effects of surface curvature and bulk depletion are likely to increasingly impact the surface of smaller aerosol particles. SFG scattering (SFS) experiments have been successfully performed on suspensions and emulsions of water droplets in oil,^76,77^ and thereby opened the door to studying small particles directly. The group of Yi Rao has worked on extending SFG scattering to free floating aqueous aerosol particles.^78−80^ While there has been some discussion about the experimental limitations involved in measuring SFS spectra of free floating aerosol,^81,82^ such experiments would promise an insight not only into the role of the particle size and geometry but may also allow to extend studies to include much more realistic systems and introducing reagents into the gasphase. In a similar vein, it would be very interesting to study, for example, suspensions of mineral particles in a liquid, since it has been shown repeatedly that decreasing the particle size of solid materials can lead to quite surprising effects. Especially an extension to studying reactions in the aerosol phase would be of great interest.
